# In vivo toxicity and antitumor activity of newly green synthesized reduced graphene oxide/silver nanocomposites

**DOI:** 10.1186/s40643-021-00400-7

**Published:** 2021-06-03

**Authors:** Mohamed M. El-Zahed, Zakaria A. Baka, Mohamed I. Abou-Dobara, Ahmed K. El-Sayed, Magy M. Aboser, Ayman Hyder

**Affiliations:** 1 Department of Botany and Microbiology, Faculty of Science, Damietta University, New Damietta, 34517 Egypt; 2 Department of Chemistry, Faculty of Science, Damietta University, New Damietta, 34517 Egypt; 3 Department of Zoology, Faculty of Science, Damietta University, New Damietta, 34517 Egypt

**Keywords:** Reduced graphene oxide, Silver nanoparticles, In vivo toxicity, Nanocomposites, Antitumor, Ehrlich ascites carcinoma

## Abstract

A novel biosynthesis of dual reduced graphene oxide/silver nanocomposites (rGO/AgNC) using the crude metabolite of *Escherichia coli* D8 (MF06257) strain and sunlight is introduced in this work. Physicochemical analysis of these rGO/AgNC revealed that they are sheet-like structures having spherically shaped silver nanoparticles (AgNPs) with an average particle size of 8 to 17 nm, and their absorption peak ranged from 350 to 450 nm. The biosynthesized rGO/AgNC were characterized by UV–vis and FT-IR spectra, X-ray diffraction, Zeta potential and transmission electron microscopy. After the injection of these nanocomposites to mice, their uptake by the kidney and liver has been proven by the ultrastructural observation and estimation of the hepatic and renal silver content. These nanocomposites caused a moderate toxicity for both organs. Changes in the liver and kidney functions and histopathological effects had been observed. The rGO/AgNC revealed a remarkable antitumor effect. They showed a dose-dependent cytotoxic effect on Ehrlich ascites carcinoma (EAC) cells in vitro. Treatment of mice bearing EAC tumors intraperitoneally with 10 mg/kg rGO/AgNC showed an antiproliferative effect on EAC cells, reduced ascites volume, and maintained mice survival. The results indicate that this green synergy of silver nanoparticles with reduced graphene oxide may have a promising potential in cancer therapy.

**Supplementary Information:**

The online version contains supplementary material available at 10.1186/s40643-021-00400-7.

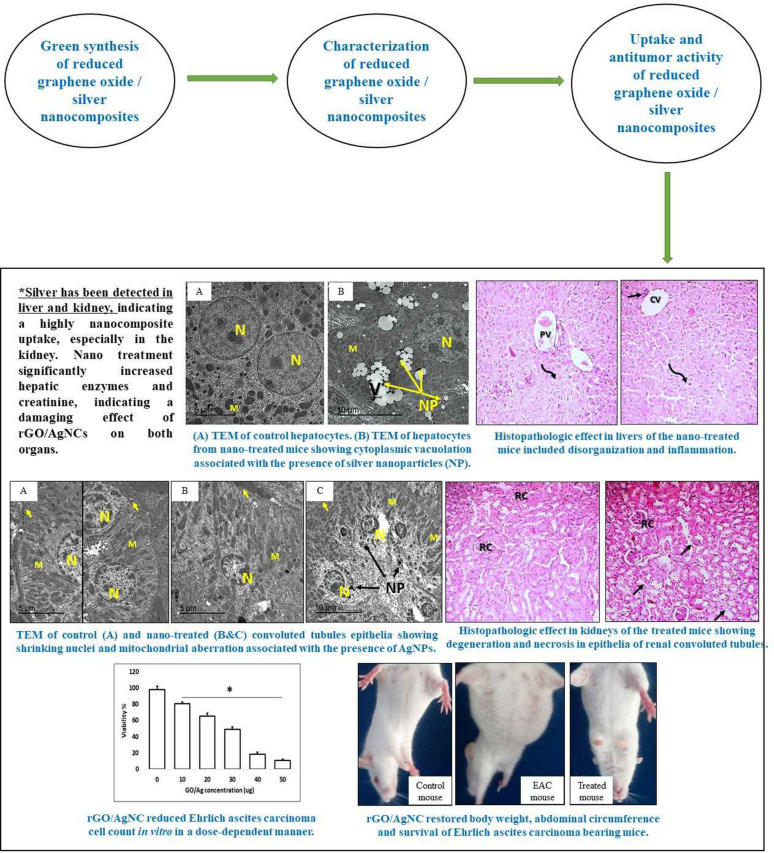

## Introduction

Graphene oxide (GO) is one of the most common carbon compounds that could be synthesized throughout the oxidizing of graphite powder. Reduced graphene oxide (rGO) compounds have been reported as antimicrobial agents (Anand et al. 2019), enhancing materials for the wound healing (Ma et al. 2019), and anticancer agents (Dhanavel et al. 2020). These activities of the rGO were referred to the presence of the functionalized groups (carbonyl (C=O), carboxyl (C–OOH), hydroxyl (C–OH), epoxy (C–O–C)) on its surface and edges (Rasoulzadehzali and Namazi 2018). Moreover, it is thought that the rGO has less oxygen-related functional group and therefore it might be good for many medical applications (Morimoto et al. 2016). Also, silver nanoparticles (AgNPs) have been used as broad-spectrum antimicrobial agents (Nasr et al. 2020) in addition to their antiangiogenesis properties (Gomathi et al. 2020). Soica et al. (2018) reported that AgNPs enhanced the anticancer activities against MCF-7 (human breast cancer) cell lines in addition to their apoptosis and the antiangiogenesis action against Ehrlich ascites carcinoma (EAC) solid tumor was recorded by El-Sonbaty (2013) and Mukherjee et al. (2014).

In past few years, scientists have given much interest and attention for the synthesis of polymer-based nanocomposites (PNC) due to their inherent advantages of biological activities comparing with the nanometallic particles or the individual state of polymers (Idumah et al. 2019). Among PNC, reduced graphene oxide/silver nanocomposites (rGO/AgNC) exhibit potent anticancer activities moreover their antimicrobial action (Pooresmaeil and Namazi 2019; Jose et al. 2020). Also, rGO/AgNC have been used in various applications including biomedical applications as wound dressing, anticancer activity and as drug delivery system (Zaidi 2019), and industrial applications as water treatment, food packaging, cosmetics, and biosensors (Ceran et al. 2020).

Green synthesis of PNC using microorganisms are considered as a better way than chemical and physical methods due to their advantageous characteristics such as safety, easy and rapid processing, stability, low cost, and flexibility (Sabayan et al. 2020). Recent studies have revealed that the crude metabolites of bacteria such as *Escherichia* and *Streptomyces* sp. act as strong bioreductant agents and could be used as microbial nano-factories (Yusof et al. 2020; Elsharawy et al. 2020).

The in vivo behavior and effect, and the biological activity of these rGO/AgNC in cancer cells have not been explored. The present study provides a green approach for the synthesis of rGO/AgNC using crude metabolite of *E. coli* D8 (AC: MF062579), their characterization, in vivo effect, and anticancer activity in mice. The presented work is, to our knowledge, a first study for the in vitro and in vivo uptake and antitumor activity of rGO/AgNC.

## Materials and methods

### Preparation of *E. coli* D8 bacterial metabolite

*Escherichia coli* D8 (AC: MF062579) used in the biosynthesis of AgNPs was obtained from our culture collection. The bacterial strain was regularly subcultured on nutrient agar plates (37 °C, 24 h.). The developed well separated colonies of bacteria were picked up and grown on nutrient broth medium (37 °C, 150 rpm, 48 h). 200 μL culture of *E. coli* D8 (0.5 McFarland standard (1–2 × 10^8^ CFU/ml)) was inoculated into 250-ml Erlenmeyer flasks containing 50 ml of nutrient broth medium (37 °C, 150 rpm, 48 h). After incubation, the bacterial metabolites were collected by centrifugation at 5000 rpm for 15 min.

### Synthesis of reduced graphene oxide/silver nanocomposites

The synthesis protocol of rGO/AgNC included the oxidation of graphite powder using strong oxidizing agents followed by reduction of GO and decoration with AgNPs. Here, the GO was prepared by oxidizing the pristine graphite powder (Loba Chemie Pvt. Ltd., India) according to Hummers and Offeman (1958) method. In brief, 2 g graphite powder was added into 50 ml of concentrated sulfuric acid in an ice-water bath and then stirred for 2 h at 35 °C. The mixture was diluted with 350 ml distilled water. Twenty ml of 30% hydrogen peroxide was added to the reaction mixture drop by drop. The resulting GO was washed out with 5% HCl solution and then with distilled water to achieve the pH of 7. The solution was centrifuged at 10,000 rpm for 15 min and dried in an oven at 60 °C for 24 h.

For the synthesis of rGO/AgNC, 0.15 g of the synthesized GO particles were dispersed in 50 mL distilled water and submitted to 2 h ultrasonication at 25 °C (ultrasonic bath, 28 kHz Delta-sonic 920 N º 484, Meaux, France). Then, 0.5 g of silver nitrate (AgNO_3_) (Panreac Quimica S.L.U, Barcelona, Spain) was added to the aqueous solution gradually and shacked well till solvation. In one step, 20 mL *E. coli* D8 crude metabolite were added as media for dual reducing procedure for both AgNO_3_ and GO in the presence of sunlight. At first, the color was turned into dark brown indicated to the formation of AgNPs then turned into dark violet-brown color indicated to the binding of AgNPs to the formed rGO. After 15 min, the rGO/AgNC powder was obtained by centrifugation at 10,000 rpm for 15 min several times and then dried in an oven at 60 °C for 24 h.

### Characterization of the synthesized graphene oxide/silver nanocomposites

Ultraviolet–visible (UV–vis) spectral analysis and Fourier transform infrared spectroscopy (FT-IR) of the formed GO and rGO/AgNC were done using UV/VIS/NIR Spectrophotometer (V-630, Japan) and FT/IR-4100typeA, respectively. The X-ray diffraction (XRD) patterns of the GO and rGO/AgNC were recorded at 2*θ* values between 10° and 80° using a Cu X-ray tube at 40 kV and 30 mA with the X-ray diffractometer (model LabX XRD-6000, Shimadzu, Japan). The Zeta Potential analysis was carried out using Malvern Zetasizer Nano-ZS90, Malvern, UK. Transmission electron microscopic analysis (TEM) was done using JEOL JEM-2100, Japan, as described before (Eldeeb et al. 2018).

### The releasability and stability of the synthesized graphene oxide/silver nanocomposites

The solubility of rGO/AgNC was tested in different solvents such as water, methanol, ethanol, dimethylformamide (DMF), *n*-butyl alcohol, acetone, toluene and hexane. The UV–vis spectral analysis of rGO/AgNC stored in dark or light was carried out using UV/VIS/NIR Spectrophotometer (V-630, Japan) and the poly dispersity index (PDI) and Zeta average size (Zavg) were calculated by the Malvern Zetasizer Nano-ZS90, Malvern, UK, according to the rate of diffusion.

The releasability of rGO/AgNC was investigated by dialysis tests. 5 ml of dispersed rGO/AgNC was added into dialysis tubes (Spectra/Por Biotech; cellulose ester; MWCO 100,000) and then submerged within 200 mL of double distilled water. Using magnetic stirrer, the dialysis experiments were stirred slowly at 37 °C. The concentration of silver ions was measured by the atomic spectrometer (PerkinElmer, PinAAcle-500, UK).

### Ehrlich ascites carcinoma cells

Transportable tumor (EAC) initial inoculum was provided by the Zoology department, Mansoura University, Egypt, in ascitic form and maintained by serial biweekly transplantations in our laboratories through intraperitoneal (i.p.) injections of 3 × 10^6^ EAC cells freshly drawn from a donor mouse after collection from peritoneal cavity, purification and counting of viable cells by trypan blue.

The cell viability test was assessed using the trypan blue cell death assay (Strober 2015). Briefly, portions of EAC cells (1 × 10^4^) were incubated with different concentrations (0–50 μg/ml) of rGO/AgNC at 37 °C for 1 h. After this incubation period, each mixture was carefully mixed with 0.4% trypan blue in 1:1 ratio and incubated for 2 min. The stained mixtures were transferred to a hemocytometer to manually cell count. The percentage of cells admitting trypan blue dye to the total number of cells was determined by counting four different fields for each experimental condition. The assay was performed in triplicates.

### Animal experiments

Female mice weighing 26–31 g were used in this study. They were housed with food and water ad libitum under standard environmental conditions of temperature and relative humidity a light/dark cycle of 12/12 h. Animal experiments have been approved by the university. They comply with the ARRIVE guidelines and were carried out in accordance with the UK. Animals (Scientific Procedures) Act, 1986 and associated guidelines, and EU Directive 2010/63/EU for animal experiments.

For studying the in vivo effect of the prepared nanocomposite, mice were intraperitoneally injected with a dose of 10 mg/kg rGO/AgNC for 7 days (Rahmanian et al. 2017). After this injection period, liver and kidney functions from the non-treated and treated groups were assessed, livers and kidneys were processed for histopathological and ultrastructural examinations, and estimation of silver content by the atomic absorption (Elsharawy et al. 2020).

For the assessment of the anticancer effect of rGO/AgNC, mice were randomly assigned to 3 groups: negative control; EAC ascites group, in which mice were inoculated with 4 × 10^6^ EAC cells; and EAC-rGO/AgNC, in which mice were inoculated with 4 × 10^6^ EAC cells, left for 1 week and then injected intraperitoneally with a dose of 10 mg/kg of rGO/AgNC daily for 7 days. Mice survival, morphological and physiological characteristics, and exudate EAC cellular content were followed.

### Chemical analyses

The mice liver and kidney samples were digested using a mixture of nitric acid (1 M) and perchloric acid (1 M) at a ratio of 2:1 (v/v) for 4 h and then incubated at 120 °C until the vaporization of the remaining acids. Samples were diluted using known volume of distilled water and then filtered for silver (Ag) total mass measurement using the atomic spectrometer (PerkinElmer, PinAAcle-500, UK).

Sera were harvested from blood samples collected from different groups and stored at -20 °C until the determination of alanine transaminase (ALT), aspartate transaminase (AST), albumin, and creatinine as described before (Eldeeb et al. 2018).

### Statistical analysis

Data were statistically analyzed using SPSS software version 25. Values were expressed as the mean ± standard deviation (SEM) and were analyzed with one-way analysis of variance (ANOVA), followed by t-test as a post hoc test. A *p* < 0.05 was considered significant in all cases.

## Results

### Characterization of the nanocomposite

**Fig. 1:**
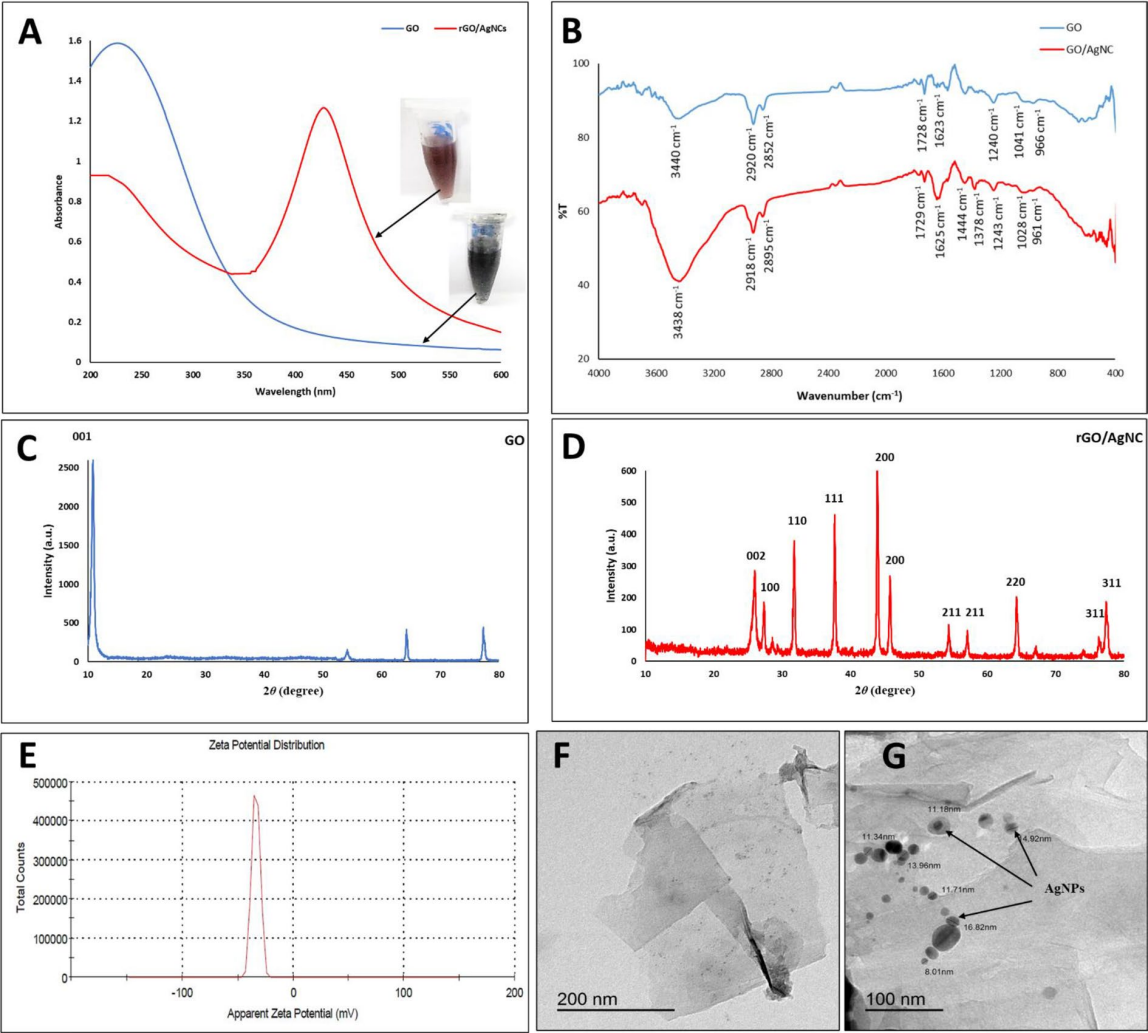
Characterization of reduced graphene oxide (GO)/silver nanocomposites (AgNC) biosynthesized by metabolite of *E. coli*. **A** The UV–vis spectra of GO and rGO/AgNC. **B** FT-IR spectra of GO and rGO/AgNC. **C** The X-ray diffraction (XRD) patterns of the GO and rGO. **D** The XRD patterns of rGO/AgNC. **E** Zeta potential measurement analysis of rGO/AgNC. **F** Transmission electron microscopy (TEM) of GO. **G** TEM of rGO/AgNC

The first indication for the formation of rGO/AgNC was the color change from black into brown. rGO/AgNC was characterized by UV–vis spectrum, FT-IR, XRD and TEM analyses. The UV–vis spectrum (Fig. 1A) of GO confirmed the π–π transitions of aromatic C–C bonds showing a peak at 230 nm (Gurunathan et al. 2015). The characteristic absorption peak for AgNPs appeared at 431 nm after their deposition on the surface of rGO. This band is assigned to the surface plasmons of AgNPs (Jebaranjitham et al. 2019).

FT-IR spectra (Fig. 1B) confirmed the characteristic peaks for GO that were observed at 3440 (OH stretching), 1728 (C=O stretching), 1623 (C=C stretching) and 1041 cm^−1^ (C–O stretching). The presence of oxygen-containing functional groups such as hydroxyl, carboxyl and epoxy proved the successfully oxidation process of graphite powder to GO (Mukheem et al. 2018). Besides, after addition and reduction of silver ions (Ag^+^), the –OH groups were stretched and shifted from 3404 to 3438 cm^−1^ that confirm the interactions between Ag^+^ and/or AgNPs and the oxygenated groups on the rGO. The identified band 1625 cm^−1^ corresponded to the functional groups (C=C) that staying on GO plates indicating to the lack of amid progressions during the oxidation processes (Yuan and Gurunathan 2017).

Figure 1C, D shows the XRD of GO and rGO/AgNC. The XRD patterns for GO exhibited the (001) characteristic peak at 2*θ* = 11.9°, which confirmed the closeness of oxygen-containing functional groups in negated spaces between graphene oxide sheets. However, this peak disappeared and a new peak at 2*θ* = 25.9° emerged indicating to the reduction of GO and the distance between its layers (Zhang et al. 2011). In addition, ten characteristic peaks of AgNPs appeared at 27.3°, 31.7°, 37.6°, 43.9°, 45.7°, 54.3°, 57°, 64.26°, 76.3° and 77.4°, corresponding to respective crystal planes (100), (110), (111), (200), (200), (211), (211), (220), (311), and (311) (Galvez et al. 2021). The Ag diffraction peaks confirmed the incidence of the face centered cubic (FCC) crystal structure on the crystalline AgNPs (Hu et al. 2013). These results confirmed the synthesis of AgNPs after reduction and then loaded on the rGO surface (Li and Liu 2010). The zeta potential analysis (Fig. 1E) showed that rGO/AgNC has a negative charge of -33.5 mV, indicating a high dispersity and stability.

The morphologies of the GO, rGO/AgNC and AgNPs samples were investigated directly by TEM. The TEM images showed the transparent and sheet-like structure of GO (Fig. 1F) and rGO/AgNC (Fig. 1G). Also, the spherical shaped and well-dispersed AgNPs were observed in the TEM and showed a mean size of 8–17 nm (Fig. 1G).

### Stability and diffusion of the nanocomposite

**Fig. 2:**
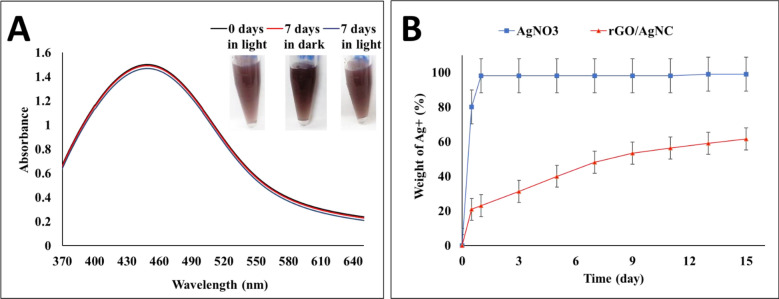
The stability and releasability of reduced graphene oxide (GO)/silver nanocomposites (AgNC) biosynthesized by metabolite of *E. coli*: **A** The UV–vis spectra of rGO/AgNC at both zero time and after storage for 7 days in dark or light conditions. **B** The release property of AgNO_3_ vs. AgNPs from rGO/AgNC at 37 °C

The rGO/AgNC showed a stable solution in the different tested solvents for 3 h. The rGO/AgNC exhibited a good dispersion in strong polar solvents such as DMF and ethanol and bad dispersion in nonpolar solvents such as toluene and hexane. These results are in agreement with previously published data (Konios et al. 2014 and Ma et al. 2018). The UV–vis spectra of rGO/AgNC did not show a significant change when stored either in dark or light for 7 days (Fig. 2A). The dialysis experiments (Fig. 2B) aimed to investigate the releasability of AgNPs from the composite. These experiments showed a completed with rapid diffusion rate of the silver ions out from the dialysis tube of AgNO_3_ within a few hours. In comparison, although present, the silver ions diffusion rate out of the dialysis tube of rGO/AgNC was very slow. The results for poly-dispersity index (PDI) and Zeta average size (Zavg) for rGO/AgNC in different solvents are shown in Additional file 1: Table S1.

### Detection of rGO/AgNC in mice organs

**Fig. 3:**
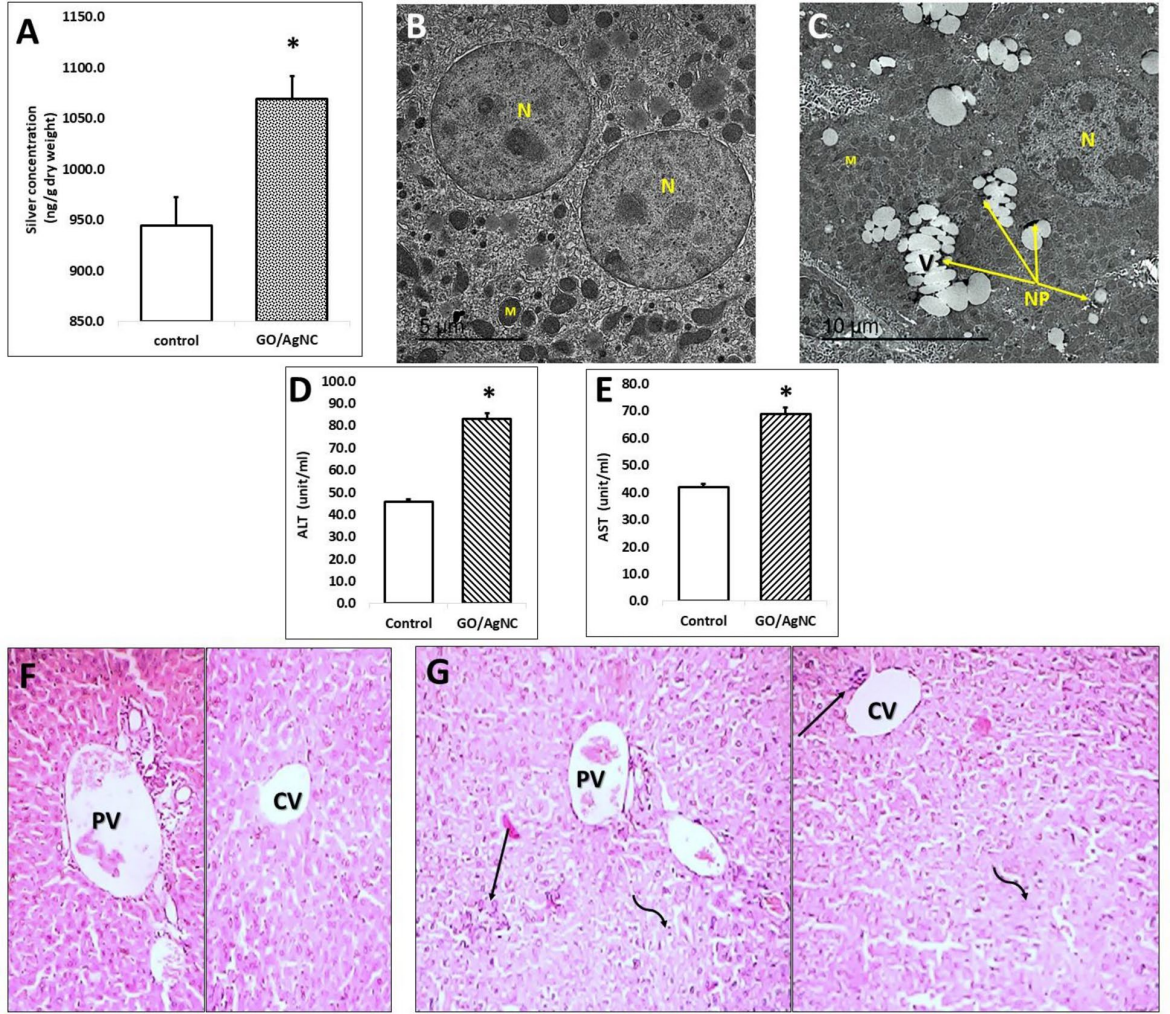
Effect of rGO/Ag nanocomposite biosynthesized by *E. coli* on mice liver. **A** silver content in the hepatic tissue. **B** TEM of control hepatocytes showing nuclei (N) and mitochondria (M). **C** TEM of hepatocytes from mice treated with rGO/AgNC showing cytoplasmic vacuolation (V) associated with the presence of silver nanoparticles (NP). Liver function was assessed by assessing serum ALT (**D**), AST (**E**) and albumin (Fig. 6D). Histopathologic effect in livers of mice treated with rGO/AgNC (**G**), compared to the control livers (**F**). Abbreviations: PV, portal vein; CV, central vein; straight arrows show mild inflammatory leucocyte infiltration; curved arrows show areas of pycnotic nuclei and focal necrosis. * denotes statistically significant different value from the control one (t-test)

**Fig. 4:**
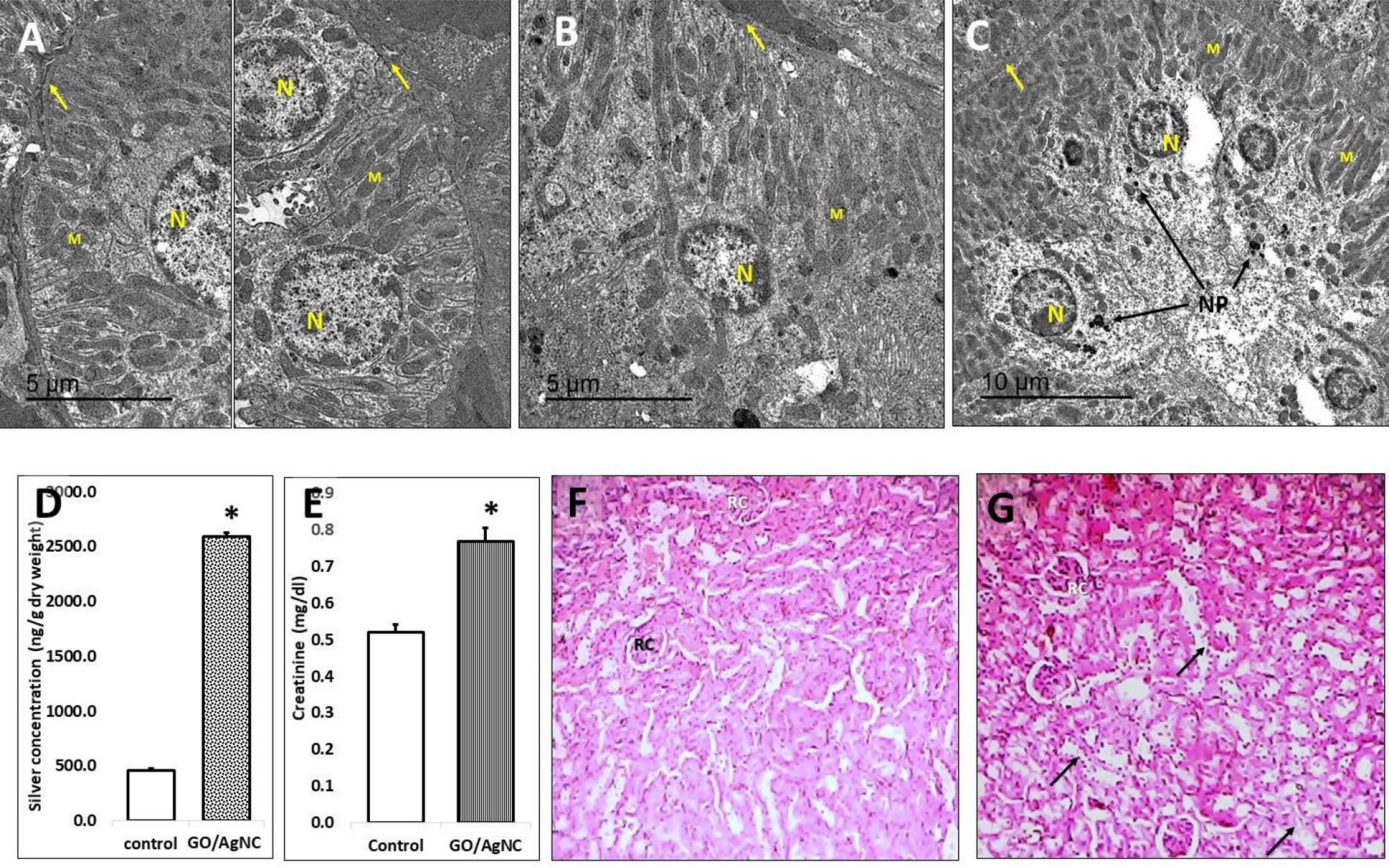
Reduced GO/Ag nanocomposites biosynthesized by *E. coli* are largely taken up by the kidney. TEM of convoluted tubules epithelia **A**, control; **B** and **C**, nanocomposite-treated) showing shrinking nuclei (N), mitochondrial aberration (M) and thickened basement membranes (arrows) in B and C, compared to that in A. **D** silver content in the renal tissue. **E** Nanocomposite treatment increases serum creatinine. The * in **D** and **E** denotes statistically significant different value from the control one (unpaired t-test). Histopathologic effect in kidneys of mice treated with rGO/AgNC (**G**), compared to the control (**F**), show dilated Bowman space of the renal corpuscles (RC) and degenerated and necrotic tubular epithelia (arrows)

Mice were treated with rGO/AgNC at a daily low dose of 10 mg/kg for a week. Results of this treatment are summarized in Figs. 2 and 3. Silver content has been estimated in both liver and kidney (Figs. 3A, 4D). The results showed a significant increase in silver content, indicating a highly nanocomposite uptake, especially in the kidney. For confirmation, both organs have been also processed for transmission electron microscopy (Fig. 3A–C), which showed deposits of nanoparticles in both organs.

### Effect of rGO/AgNC on mice

#### Effect of rGO/AgNC on the ultrastructure of the liver and kidney

Transmission electron micrograph examination of control mice hepatocytes (Fig. 3B) revealed normal ultrastructural architecture of the hepatocytes. The nucleus had a double membrane of the nuclear envelopes with nuclear pores. Heterochromatin dominated the peripheral nuclear areas and interchromatin granule clusters. The nucleolus was composed of dense fibrillar component, granular component and a fibrillar center, and accompanied by masses of heterochromatins. Golgi apparatus, mitochondria with cristae, outer and inner membranes, smooth endoplasmic reticulum, rough endoplasmic reticulum with ribosomes, lysosome, microbodies, and glycogen granules appeared normal.

Transmission electron micrograph examination of hepatocytes of mice treated with rGO/AgNC (Fig. 3C) revealed ultrastructural lesion in hepatocytes, which showed nucleus with disrupted organization of nuclear components as clumped heterochromatin and wrinkled nuclear membrane. The prominent feature lesion showed in cytoplasm was the electron lucent vacuolation adjacent to NP-containing endosomes. The mitochondrial abnormality was represented by rarely observed cristae and increased matrical substance. The observed few tiles of rough endoplasmic reticulum devoid ribosomes. Many ribosomes were recognized in cytoplasm.

Examination of transmission electron micrograph of control mouse renal tissue (Fig. 4A) revealed normal ultrastructural architecture of the proximal convoluted cuboidal epithelial cells. The large heterochromatic nucleus is located more toward the base than the apex of cell; many vacuoles distributed throughout the apical area of the cell; lysosomes, Golgi apparatus and apical microvilli and small endocytotic vesicles that have pinched off from the plasma membrane at the base of the microvilli showed normal appearance. The prominent feature observed in the cell of proximal convoluted epithelium is the extensive numbers of longitudinally oriented mitochondria presented in the cell within long invaginating folds of cell membrane, which caused basal striations appearance. The extreme basal aspect of these invaginating folds revealed a dense material that represents bundles of actin filaments. The electron micrograph also revealed thin basement membranes of the tubule, basal lamina and a small amount of connective tissue and the fenestrated endothelium of an adjacent peritubular capillary.

Examination of transmission electron micrograph of renal tissue from mice treated with rGO/AgNC (Fig. 4B, C) revealed ultrastructural lesions in the cuboidal cells of the proximal convoluted epithelium, which had shrunken nuclei with clumped heterochromatin. The mitochondrial aberration represented by rarely observed cristae, and the basal striations appearance are disorganized. The epithelial cells had thickened basement membranes of the tubule. The important lesion feature observed in the micrograph is represented by dense deposits of NPs distributed in many places and causing cytoplasmic distortion (Fig. 4C).

#### Histopathological effect of rGO/AgNC on the liver and kidney tissues

Histological examination of liver from the control mice (Fig. 3F) revealed normal hepatic architecture. The hepatic parenchyma consisted of hepatocytes arranged in cellular plates radiating from the central vein at the center of hepatic lobules and several sets of blood vessels at its periphery. The peripheral vessels are grouped in connective tissue of the portal tracts and include a branch of the portal vein, a branch of the hepatic artery, and a branch of the bile duct (the portal triad). Both blood vessels in this triad branched as sinusoids, which ran between plates of hepatocytes.

Histopathological examination of liver revealed lesion associated with the treatment of rGO/AgNC (Fig. 3G). This lesion is characterized by disorganization of hepatic lobular structure, foci aggregation of inflammatory cells in the hepatic parenchyma surrounded by shrunken deeply acidophilic hepatocytes, periportal mild inflammatory cellular infiltration, degenerated and necrotic hepatocytes and some inflammatory cellular infiltration surrounded the central vein.

Renal histological section examination of control (Fig. 4F) revealed normal renal architecture. The renal parenchyma consisted of network of closely packed, proximal and distal convoluted renal tubules lined by acidophilic cuboidal cells, sectioned randomly in different planes. Spherical renal corpuscles are interspersed between renal tubules, each corpuscle contained glomerular capillary tuft that is surrounded by visceral and parietal epithelial layers of Bowman’s capsule.

Examination of renal histological section from mice treated with rGO/AgNC (Fig. 4G) revealed degeneration and necrosis in cellular epithelia of proximal and distal renal convoluted tubules. The renal corpuscles appeared diminished and distorted with dilated Bowman space and bilobed or shrunk glomerulus.

#### Liver and kidney function

The mice liver and kidney functions were tested by measuring ALT (Fig. 3D), AST (Fig. 3E), and creatinine (Fig. 4E) after treatment with rGO/AgNC. The results revealed that the nano-treatment significantly increased both hepatic enzymes ALT and AST as well as creatinine, indicating a damaging effect of rGO/AgNC on both organs.

Taken together, ultrastructural, histopathological, and biochemical examinations confirm that the green synthesized reduced graphene oxide/silver nanocomposite exerts a toxic effect in mice.

### Anticancer activity of the rGO/AgNC

#### In vitro* cytotoxic effect of rGO/AgNC*

**Fig. 5:**
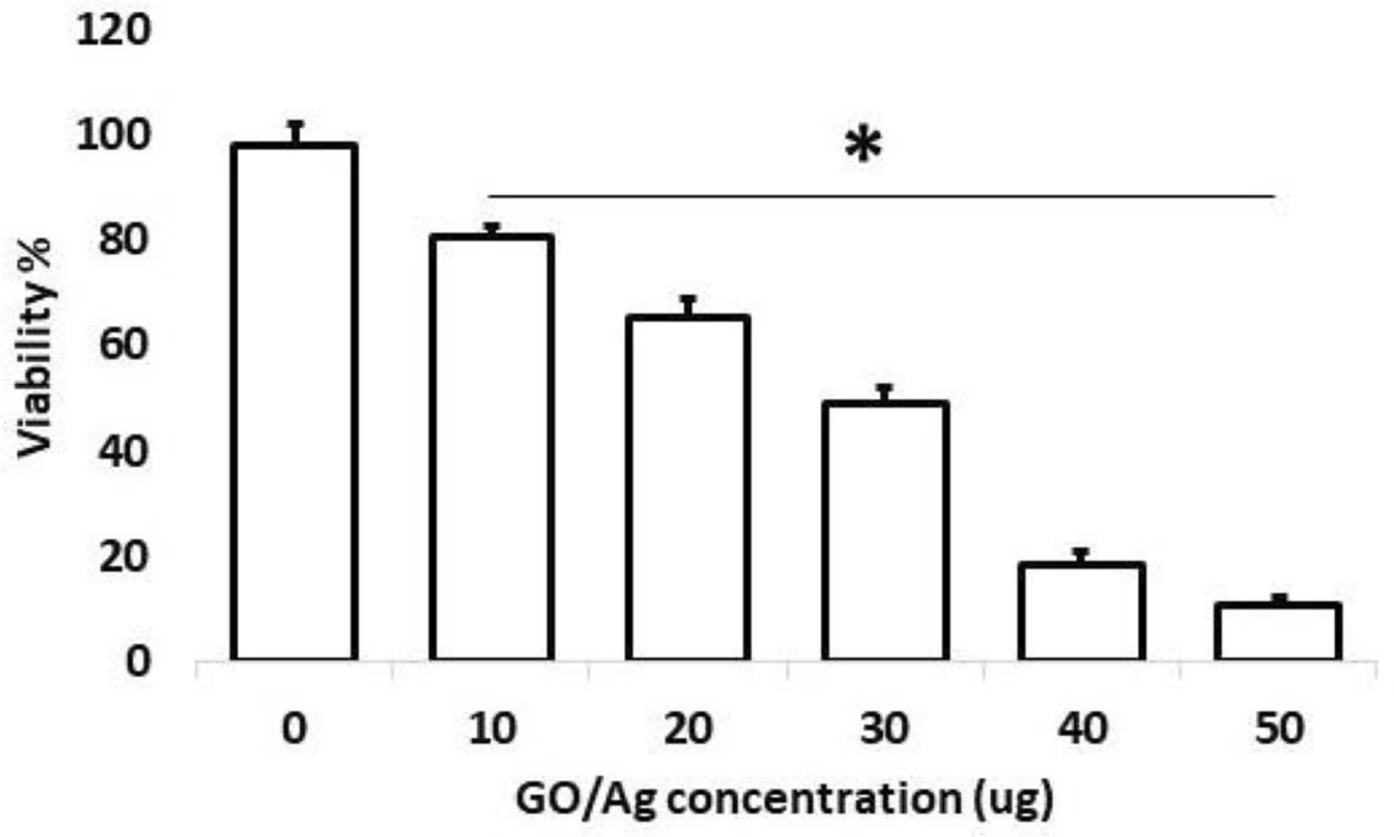
In vitro toxicity of rGO/AgNC in Ehrlich ascites carcinoma cells. Cells were incubated with different concentrations of the nanocomposite for 1 h and viability was determined by trypan blue. * denotes significantly different values from the control one (ANOVA *p* < 0.05)

For this purpose, EAC cells were grown in mice for a week and then isolated from their peritoneal exudates. These cells were incubated with different concentration of rGO/AgNC as shown in Fig. 5 for an hour and then processed for vital staining via trypan blue exclusion. The results showed that all used rGO/AgNC concentrations significantly reduced the viability of EAC cells in a concentration gradient manner, so that 50 μg/mL of rGO/AgNC could be able to kill about 87% in 1 h (Fig. 5).

### Antitumor effect of rGO/AgNC in vivo

**Fig. 6:**
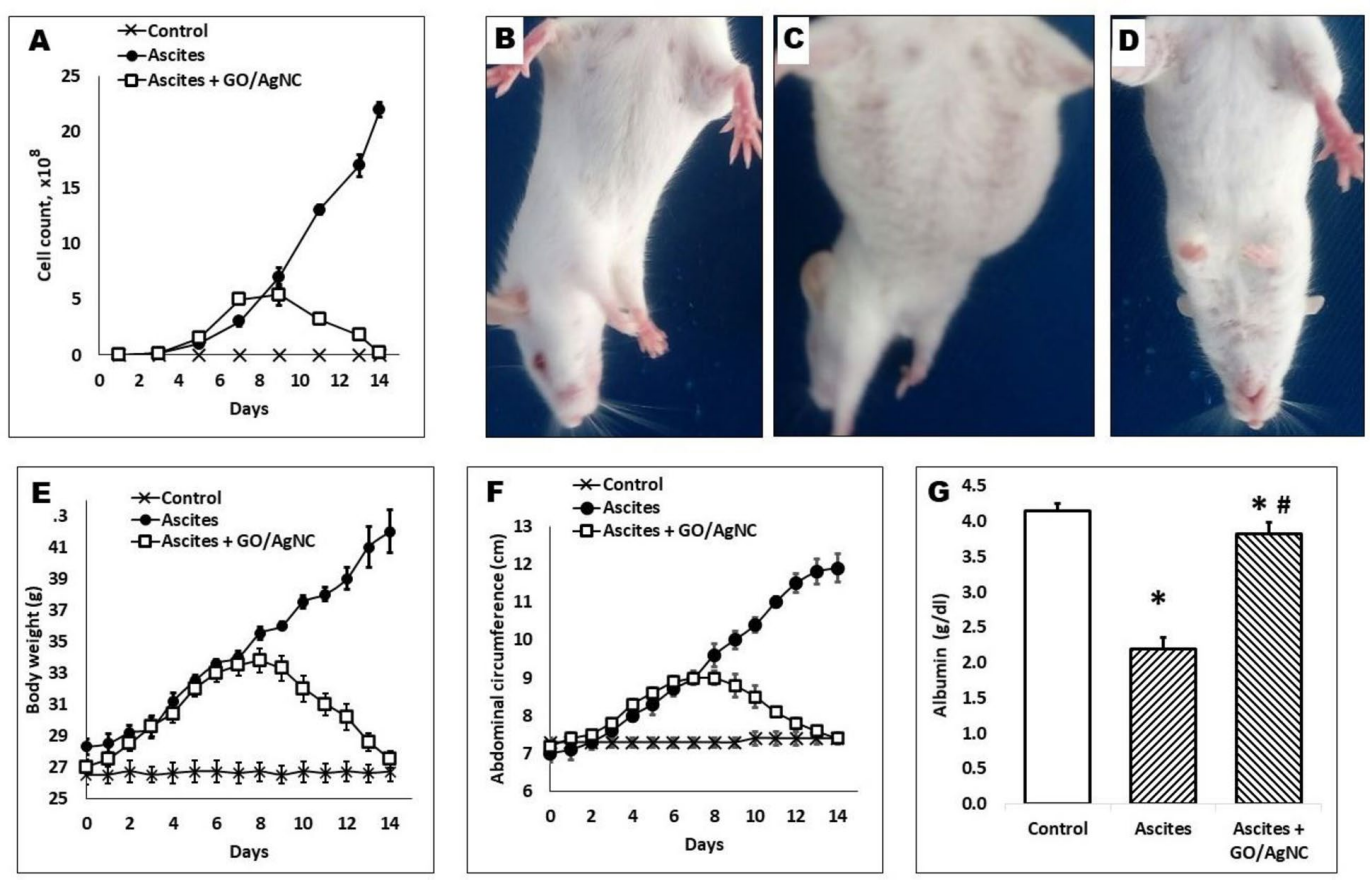
Antitumor effect of rGO/AgNC in vivo. **A** Daily reductions in Ehrlich ascites carcinoma (EAC) cells in the peritoneal exudate of mice treated with 10 mg/kg rGO/AgNC for 7 days, a week after initial 4 × 10^6^ EAC cell injection. **B**–**D** The nanocomposite reduces the ascites volume in EAC-bearing mice: **B** control mice, **C** mice injected initially with EAC cells, and **D** EAC-bearing mice treated daily with rGO/AgNC. **E**, **F** Restoration of the body weight and abdominal circumference in EAC-bearing mice after rGO/AgNC treatment. **G** Treatment with rGO/AgNC restores serum albumin level (ANOVA *p* < 0.05, * and # denote significantly different values, as determined by post hoc t-test, from the control and EAC-bearing group, respectively)

EAC cells were i.p. transplanted into mice and left to grow for a week before the daily i.p. injection of 10 mg/kg rGO/AgNC for another week. The results summarized in Fig. 6 reflected a clear regression of the ascites carcinoma and restoration of mice morphology and physiology. EAC cells from the peritoneal cavity were stained and counted every second day and showed daily reduction in their count after rGO/AgNC treatment (Fig. 6A). The nanocomposite-caused reduction in tumor cells restored the abdominal volume, circumference, and body weight in EAC-bearing mice. The drop of the ascites volume restored the blood albumin level (Fig. 6G). As a result of this rGO/AgNC anticancer activity, no mortality was recorded in EAC-bearing mice for the following 60 days (n = 9).

## Discussion

During the last decades, rGO and AgNPs deserved an enormous academic interest and a significant attention especially as apoptotic agents (Zhang et al. 2011, 2020). There were different methods that used reducing chemical agents in the synthesis of rGO/AgNC such as poly(*N*-vinyl-2-pyrrolidone) (Hu et al. 2013) and ammonia (Chook et al. 2012). Bao et al. (2011) prepared the rGO/AgNC using AgNO_3_ as a salt precursor, hydroquinone as the reductant, and citrate as the stabilizer. Pasricha et al. (2009) synthesized the rGO/AgNC under alkaline conditions using sodium borohydride as a reducing agent to Ag^+^. In addition, different physical methods were used in the synthesis of rGO/AgNC, such as that of Zainy et al. (2012) who studied the preparation of rGO/AgNC via rapid thermal treatment. They used silver acetate as a silver precursor and GO served as a substrate for the AgNPs that treated at 1000 °C in a furnace for 20 s under an ambient atmosphere. On the other hand, the green synthesis approach of nanomaterials possesses minimum toxicity, lower-cost, lower reaction temperature and lower reaction time compared to the other physical and chemical methods (Awwad et al. 2020). Gurunathan et al. (2015) reported the synthesis of rGO/AgNC in three separated steps starting with synthesis of AgNPs using *Tilia amurensis* leaves extract, reduction of GO by *T. amurensis* leaves extract and then mixing the rGO with AgNO_3_ to synthesized rGO/AgNC. Thus, the exploitation of green synthesized silver nanomaterials had an increasing interest in the elaboration of safe bioactive biomaterials in addition to their distinguished properties, such as antimicrobial activity, antiviral and antiangiogenesis action (Patil et al. 2019; Galvez et al. 2021). The present work provided a one-step green method for the synthesis of rGO/AgNC by dual bioreducing of GO and Ag in the presence of sunlight and revealed promising effect of this nanocomposite in of cancer treatment. In addition, it explored the in vivo toxicity of this nanocomposite, and revealed a moderate effect on mice liver and kidney.

The results demonstrate that the synthesized nanocomposite is stable to a great extent, with a slow release of the AgNPs, which is in consistence with the observed uptake and moderate toxicity of AgNPs by the mice tissues in subsequent experiments. In general, when the solute disperses in a solvent with a small poly dispersity index (PDI) and Zeta average size (Zavg), it denotes to the good dispersion of the solute in the solvent. The Zeta Potential analysis added to the characterization of the reduced graphene oxide/silver nanocomposite (rGO/AgNC). The solubility of rGO/AgNC were tested in different solvents. The UV–vis spectral analysis of rGO/AgNC stored in either dark or light were carried out, and the PDI and Zavg were calculated according to the rate of diffusion. The size distribution range of rGO/AgNC is highly homogenous as confirmed by measuring nanocolloidal solution PDI, which value was in the size distribution homogenous range (0–1). Studies have shown that the size, morphology, stability, and properties of the nanomaterials are influenced by the experimental conditions, the kinetics of interaction of ions with reducing agents, and adsorption processes of a stabilizing agent on the nanomaterials surface (Danaei et al. 2018; Narayanan et al. 2020). The recorded results showed that the rGO/AgNC formed a uniform dispersion in water, DMF and n-butyl alcohol due to the PDI was smaller than 0.5 d.nm and Zavg sizes was smaller than 400 nm. In addition, the high negative potential of the rGO/AgNC (− 33.5 mV) confirmed the high dispersity, good colloidal nature, and long-term stability without any aggregation.

The main parameters of rGO/AgNC are its purity, shape and size which control its biological activities (Nel et al. 2006). The obtained data by UV–vis spectroscopy, FT-IR, XRD and TEM studies confirmed the crystalline nature of AgNPs and strong interactions between the AgNPs and rGO. The TEM micrographs showed the transparent and sheet-like structure of rGO/AgNC embedded with spherical shaped and well-dispersed AgNPs. These observations matched with that of Cobos et al. (2020) study in which the obtained GO/AgNC sheets via the in situ method through the simultaneous reduction of AgNO_3_ and GO using l-ascorbic acid showed flexible sheets, paper-like structures morphologies of GO with few layers. The dark areas showed the thick stacking nanostructure of several GO and rGO/AgNC layers. The lower opaque areas designate much thinner sheets of a GO and rGO/AgNC layers indicating to their delamination. This exfoliation might increase the surface area of the synthesized materials (Stobinski et al. 2014). The resulting rGO/AgNC in the presented study showed the decorated AgNPs had an average size of 8–17 nm on the rGO sheets. Chook et al. (2012) fabricated GO/AgNC using microwave irradiation with 40.7 ± 7.5 nm of AgNPs on the GO sheets. Yun et al. (2013) prepared GO/AgNC with deposited AgNPs with an average size of 2 to 4 nm.

There are many hypotheses for mechanisms of rGO/AgNC formation but the more likely is the AgNPs were bound to GO through the collaboration of the Ag^+^ with the oxygenized functional groups on the GO surface such as the hydroxyl, epoxy and carboxylic groups (Faria et al. 2012). El-Dein et al. (2021) confirmed the presence of proteins in the biosynthesis of AgNPs by *E. coli* D8 as stabilizing and capping agents for AgNPs. The protein capping agents around the AgNPs might interact with GO-oxygenated groups as de Faria et al. (2014) supposed that as a probable mechanism of the biosynthesis of GO/AgNC which may be similar to our involved mechanism for the synthesis of rGO/AgNC.

Ehrlich carcinoma has a closeness with human tumors and is considered the most sensitive type to chemotherapy. EAC cells are undifferentiated cancer that are primordially hyperdiploid and does not have tumor-specific transplantation antigen. In addition, they have rapid proliferation, short life span as well as high transplantable capability (Ozaslan et al. 2011). In the present study, rGO/AgNC reduced EAC cell count in vitro in a dose-dependent manner. The potential in vivo toxicity of nanomaterials is always considered as a great concern for using in the biomedicine applications. The formation of solid tumors and the ascites elevate weight and abdominal circumference in mice and decrease the survival time (Ninomiya et al. 2009). The treatment with some chemicals decreased the amount of ascitic fluid without notably inspiring the number of tumor cells (Sugiura 1958). In this study, treating Ehrlich carcinoma bearing mice with rGO/AgNC at the dose of 10 mg/kg for 7 days could prolong the survival for more than 60 days, while the rGO/AgNC-untreated ones were all died within 3 weeks. In addition, treatment with rGO/AgNC restored body weight, abdominal circumference due to the reduction of the carcinoma cell viability and consequently of the ascitic fluid volume.

The penetration and accumulation of AgNPs in the treated mice were confirmed using histological and TEM examinations of liver and kidney. One of the main functions of the liver is to remove hazard compounds from the blood and transform those to suitable chemical forms that can be excreted by the kidney. Therefore, liver and kidney are the most prominent targets of nanoparticles. However, the observation of AgNPs in both organs infers the release of silver nanoparticles from the rGO/Ag nanocomposite. rGO/AgNC is formed of two parts: the rGO scaffolds and the decorated AgNPs. Polycationic rGO scaffolds could interact with the cell membrane negatively charged components (Ruiz-Herrera et al. 2006). It was reported that this interaction leads to the transposition of the potassium ions on the cell surface and losing of ionic equilibrium, which prompts the further efflux of potassium ions from the cell. This efflux leads to the hyperpolarization of the plasma membrane. It was affirmed that the plasma membrane hyperpolarization resort to the cell to increase the uptake of cations to balance the membrane potential (Peña et al. 2013). Vazquez-Muñoz et al. (2014) confirmed the gradual release and the spontaneous ionization of the Ag^+^ from AgNPs. These ions penetrate the cell throughout a cationic influx. After Ag^+^ entering the cells, different Ag related toxic effects may result as observed in this work. This observed toxic effects of AgNPs are in agreement with that of many previous reports (Hajipour et al. 2020; Elsharawy et al. 2020).

In the present study, the efficacy of the prepared nanocomposite was assessed in vivo by following up the change in the intraperitoneal Ehrlich ascites (fluid) tumor volume and cellular viability after intraperitoneal treatment with the nanocomposite, and a marked inhibition in Ehrlich tumor growth was demonstrated. This marked decrease in Ehrlich tumor volume upon treatment can be explained by the enhanced permeability and retention (EPR) effect. The EPR effect is based on the hyperpermeability of the tumor vessels due to the large holes between endothelial cells of the tumor vessel wall, and the absence of lymphatics, allowing therapeutic particles to enter the tumor and stay there (Jain and Stylianopoulos 2010). In our case, both tumor and the therapeutic nanocomposite were present together in the peritoneal cavity and retained there for the entire treatment period. These are ideal conditions for the EPR effect. However, this fluid Ehrlich tumor model cannot answer the question about the delivery of our rGO flakes that exceed 300 nm to solid tumors, where the effect of the nanoparticle size must be considered for optimal drug delivery and therapeutic outcome. Nevertheless, it is known that the holes in the solid tumor vessel wall range from 200 nm to 2 μm with an average of about 400 nm (Gao et al. 2012), compared to holes of the normal vessel wall with size of less than 10 nm. In this context, our prepared nanocomposite would be advantageous since it could selectively pass through the holes of the solid tumor vessels but will not be able to extravasate to normal tissues, reducing the adverse toxic effects. Also, the negative charge of this nanocomposite may prevent from being recognized and prolonged their circulation time in blood, which in turn enhanced the EPR effect (Pei et al. 2020). Therefore, in this study, the size and charge of rGO/AgNC were suitable for the EPR effect in the studied model and can be advantageous for application in treatment of solid tumors.

Traditional cancer therapies are disadvantaged by the damage of neighboring healthy cells and any dividing stem cell, instability of drugs, acquired resistance and toxicities, and inadequate dosage at tumor sites. Therefore, newer policies are always required to overcome these problems. Recently, several studies have established promising advantageous aspects using graphene-based composites in anticancer therapy, including selectivity and biocompatibility, dual-drug delivery system, and safety profile towards normal cell (Novoselov et al. 2012; Some et al. 2014; Saikia et al. 2016; George et al. 2018; Choi et al. 2018; Jose et al. 2020). However, the development of these composites to the next in vivo and clinical stages remains rare and slow.

This present study demonstrated that the combination of rGO and AgNPs had many advantages to the field of anticancer therapy such as controlling drug release due to the long-term stability of the nanocomposite, enhancing therapeutic efficacy, cost effectiveness, retaining the AgNPs well dispersed. These advantages were accompanied by minimal systemic toxicity. The graphene will increase the surface area, hence decreasing the toxicity of the nanometal (Alsharaeh et al. 2017). These nanocomposites cannot be taken up by normal tissues due to their large size and would be directed preferentially to the tumor tissue due to the EPR effect. However, the release of silver nanoparticles was observed in tissues and proven experimentally. These tiny AgNPs may cause toxicity. This toxicity is the main factor for the antitumor activity, but a minimal to moderate long-term toxicity in normal tissue is observed in this study and not excluded, depending on the quantity and speed of AgNP release. Studies have shown that the size, morphology, stability, and properties of the nanomaterials are influenced by the experimental conditions, the kinetics of interaction of ions with reducing agents, and adsorption processes of a stabilizing agent on the nanomaterials surface (Danaei et al. 2018). Experiments for the optimization of doses and controlling the AgNPs releasability are still required.

## Conclusions

The present study demonstrated a green, economic, simple one-step biosynthesis method for rGO/Ag nanocomposite production using the crude metabolite of *Escherichia coli* D8 (MF06257) and sunlight. The formed rGO/AgNC sheets were decorated with well dispersed spherical shaped AgNPs. Although these rGO/AgNC showed moderate toxicity in mice, they revealed a strong antitumor effect.

### Supplementary Information

**Additional file 1: Table S1.** The poly-dispersity index (PDI) and Zeta average size (Zavg) for rGO/AgNC in different solvents.

## Data Availability

The datasets used and/or analyzed during the current study are available from the corresponding author upon request.

## Abbreviations

rGO/AgNC: Reduced graphene oxide/silver nanocomposites
EAC: Ehrlich ascites carcinoma
GO: Graphene oxide
rGO: Reduced graphene oxide
AgNPs: Silver nanoparticles
PNC: Polymer-based nanocomposites
UV–vis: Ultraviolet–visible
FT-IR: Fourier transform infrared spectroscopy
XRD: The X-ray diffraction
DMF: Dimethylformamide
PDI: Poly dispersity index
Zavg: Zeta average size
EPR: Enhanced permeability and retention
i.p.: Intraperitoneal
ALT: Alanine transaminase
AST: Aspartate transaminase
