# Supplementary material for: In vivo toxicity and antitumor activity of newly green synthesized reduced graphene oxide/silver nanocomposites
Source: Bioresour Bioprocess. 2021 Jun 3;8(1):44. doi: 10.1186/s40643-021-00400-7 (PMC10992821; doi:10.1186/s40643-021-00400-7)
Supplement: Supplementary file 1 — Additional file 1: Table S1. The poly-dispersity index (PDI) and Zeta average size (Zavg) for rGO/AgNC in different solvents. [file 40643_2021_400_MOESM1_ESM.docx]

**Additional file 1**

**Table S1.** The poly dispersity index (PDI) and Zeta average size (Zavg) for rGO/AgNC in different solvents

| **Solvent** | **PDI (d.nm)** | **Zavg (nm)** |
| --- | --- | --- |
| **Water** | 0.421 | 324 |
| **Methanol** | 0.8 | 850 |
| **Ethanol** | 0.7 | 471 |
| **DMF** | 0.5 | 397 |
| **n-butyl alcohol** | 0.5 | 336 |
| **Acetone** | 0.8 | 1643 |
| **Toluene** | 0.9 | 1177 |
| **Hexane** | 0.6 | 632 |
